# Scarlatina1Read at the thirty-first annual meeting of the Medical Association of Central New York, at Auburn, October 18, 1898.

**Published:** 1899-03

**Authors:** A. A. Young

**Affiliations:** Newark, N. Y.


### SCARLATINA.[1]

BY A. A. YOUNG, M. D., Newark, N. Y.

SCARLATINA cannot be regarded in any other light than that it is a communicable disease conveyed from person to person, though the "how or which" is yet to be determined.

That the disease is produced by the development of a previously existing bacillus seems rational for, by this method is life, as seen through material forms, reproduced, it is not conceivable that antimated forms originate *de novo*. Taking this as a self-evident fact it is easy to infer that all bacterial diseases, especially the contagious ones, are the resultants of the developing specific bacillæ in the blood and that the ocular manifestations, such as the membrane of diphtheria, the eruptions of small-pox, scarlatina and the like are but the niduses of the fully developed bacillæ.

The simple presence of the fully developed bacillæ does not constitute disease; they first become active and start the processes of reproduction by depositing the germicidal vesicles or spores from which the bacillæ are to be developed, in some suitable culture medium of the body, and it is during their process of growth that pathological conditions are produced that constitute disease. If this theory be tenable then it is reasonable to direct our efforts toward the immunising of our patients, or to render inert and worthless such substances as are required for the development or growth of specific bacillæ. This has been accomplished in a few cases, the mos

1. Read at the thirty-first annual meeting of the Medical Association of Central New York, at Auburn, October 18, 1898.

notable of which is vaccination for the prevention of small-pox. That some diseases have been modified and shorn of their terrors by the immunising treatment can no longer be questioned by observant and thinking men; this treatment, then, seems to be the rational one so far as it can be practically and intelligently carried out.

In scarlatina, then, first comes nausea and vomiting, chills followed by high fever, which marks the onset of the disease, the beginning of bacillæ development; some time following this comes the peculiar eruption of the skin, which marks the time of completion and deposition of the fully developed bacillæ within these dermal patches and there they remain inert until a suitable menstruum be found in which they can complete another biological cycle; their destruction in and elimination from their home is to prevent in a measure a reinfection. Unfortunately, antiseptics administered internally are of little service, for what will destroy the developing bacillæ by contact is capable of destroying many elements on which human life depends.

The scarlatina bacillus yet remains to be positively demonstrated, but that such bacillus exists seems to admit of no doubt. Under our present knowledge we must watch and wait for its positive demonstration and be content with our empyrical and symptomatic treatment.

The condition of the digestive organs, as indicated by the appearance of the tongue, the deposit upon the tonsils, the high fever, the dry and itchy skin, followed by the peculiar rash, claim our attention in formulating a treatment, not the treatment, for this must be based upon a correct etiology and pathology. The specific germ must first be known, its methods of growth fully understood and a knowledge of the agent that will prove its destroyer must first be acquired before the disease can be successfully and intelligently treated.

The medicinal agents which seem to be indicated and which have served a good purpose, given cautiously, are the coal-tar derivatives, combined with pilocarpin, aconite and some of the simple bitter tonics. This treatment might be termed the eliminative one and of these agents pilocarpin may be regarded as par excellence; it will stimulate to action all of the excretory organs of the body with but little depressing influence as an after-effect. With the administration of a single dose, say one-fourth grain, as a rule there soon follows intense salivation and sweating, with marked increase of excretion of urine and as a result a rapid decline in temperature and a general feeling of relief. With the free use of this agent, which covers a period of over fourteen years, the writer has yet to observe the first instance where it exerted a deleterious influence over the heart's action. It seems, therefore, that the prevalent opinion that it is a powerful heart depressant is based upon theory instead of practical observation. It is a drug that promises much as an eliminating agent in infectious fevers; it does its work by the process of elimination, either of the developing bacillæ or of the substances on which they feed ; it may be a starving process.

So far as the coal-tar derivatives are concerned, they are administered for only one purpose, viz., to quiet cerebral disturbances over which in some unknown way they have a marked influence..

After the initial symptoms have subsided and temperature approaches the normal, further restorative measures are largely left to nature, which she manipulates well if left unhampered by active medicinal agents.

A word now concerning the diatetic treatment and a word covers almost the entire ground. At the onset the patient usually wants nothing save water, which should be given in abundance. Why burden the system with food, causing more work which means more waste of vital forces when to the patient food is actually repulsive ?

The disease is self-limited ; starvation cannot occur, though no food be taken during the entire run of this specific fever ; follow out, therefore, the patient's wishes and desires ; his nature knows best what he needs in this direction, knows best what is needed to supply the demand caused by scarlatinal wastefulness. It is not a case like the starvation of typhoid at convalescence, where the patient desires everything and anything, and human judgment must then interfere. The normal craving of the system is all we meet with, which is an index of what the supply should be. The present treatment, then, would to be one that would reduce the abnormal conditions to a minimum, after which following out the expectant plan—expect the patient to recover.

The final act, the period of desquamation, requires the most careful attention, for the propagation of the disease is largely, if not entirely, upon the spores existing in the exfoliated dermal patches. Thanks to the smoked-ham rind of our fathers, they builded better than they knew. The more modern therapeutic agents, carbolised oil and vaseline, are not the peers of their predecessors, the former having the more "penetrating" power, while the latter succeed better in hermetically sealing and preserving the vitality of the spores within their nidus—the scale.

Why use oils at all? There is a far better method theoretically, and one, the practice of which, indicates the theory to be a fact. In formaldehyde we seem to have the ideal germicide; this agent possesses hardening, penetrative and germicidal properties found in no other drug, which makes it especially valuable for sterilising the epithelial scales, which doubtless contain the germinal spores. It matters but little, then, whither they may drift, for from them similar life cannot spring without a new infection, and it is probable that the scales are so altered by the action of this agent upon them that they may never again become germ propagators. Formaldehyde approaches the ideal germicide and as such its use during the desquamative stage of scarlatina is markedly indicated, and is commended.

22 East Miller Street.

